# Βio-Based Epoxy/Amine Reinforced with Reduced Graphene Oxide (rGO) or GLYMO-rGO: Study of Curing Kinetics, Mechanical Properties, Lamination and Bonding Performance

**DOI:** 10.3390/nano12020222

**Published:** 2022-01-10

**Authors:** Sheikh Rehman, Julio Gomez, Elvira Villaro, Dwane Cossey, Panagiotis G. Karagiannidis

**Affiliations:** 1 School of Engineering, Faculty of Technology, University of Sunderland, Sunderland SR6 0DD, UK; bh31lt@research.sunderland.ac.uk (S.R.); bg72yy@student.sunderland.ac.uk (D.C.); 2 Avanzare Innovacion Tecnologica S.L., Av. Lentiscares 4-6, 26370 Navarrete, Spain; julio@avanzare.es; 3 Instituto de Tecnologías Químicas de La Rioja (Inter-Química), San Francisco 11, 26370 Navarrete, Spain; evillaro@interquimica.org

**Keywords:** nanocomposites, reduced graphene oxide, curing kinetics, differential scanning calorimetry, lamination, bonding, mechanical properties

## Abstract

In this work, we report the synthesis and study of nanocomposites with a biobased epoxy/amine (Epilok 60-600G/Curamine 30-952) matrix reinforced with reduced graphene oxide (rGO) or functionalised with 3-glycidoxypropyltrimethoxysilane (GLYMO-rGO). These graphene related materials (GRMs) were first dispersed into a Curamine hardener using bath ultrasonication, followed by the addition of epoxy resin. Curing kinetics were studied by DSC under non-isothermal and isothermal conditions. The addition of 1.5 wt% of GLYMO-rGO into the epoxy matrix was found to increase the degree of cure by up to 12% and glass transition temperature by 14 °C. Mechanical testing showed that the addition of 0.05 wt% GLYMO-rGO improves Young’s modulus and tensile strength by 60% and 16%, respectively, compared to neat epoxy. Carbon fibre reinforced polymer (CFRP) laminates were prepared via hand lay up, using the nanocomposite system GRM/Epilok/Curamine as matrix, and were cut as CFRP adherents for lap shear joints. GRM/Epilok/Curamine was also used as adhesive to bond CFRP/CFRP and CFRP/aluminium adherents. The addition of 0.1 wt% GLYMO-rGO into the adhesive and CRFP adherents showed improved lap shear strength by 23.6% compared to neat resin, while in the case of CFRP/Aluminium joints the increase was 21.2%.

## 1. Introduction

The need to reduce fuel consumption and carbon emissions in the transport industry urges towards multi-material design strategies to maximise weight reduction [1]. Many parts of vehicles where heavy metals are used could be replaced with polymer-matrix composite materials, which offer good mechanical properties at reduced weight, lowering fuel consumption at the use phase [2]. It has been estimated that every 100 kg reduction in weight reduces fuel consumption by 0.4 L/100 km [3]. The transition to electric vehicles still necessitates lightweighting, as the heavy battery packs increase vehicle weight and energy consumption.

Carbon fibre reinforced polymer (CFRP) composite materials comprise carbon fibres as the strong and stiff reinforcing agent, combined with a tough polymer matrix, which is typically a thermoset polymer. One of the main thermosets that dominate the CFRPs market are epoxies; a class of high-performance crosslinked polymers with a broad range of applications, e.g., paints and coatings, adhesives and composite matrix [4].

**Figure 1:**
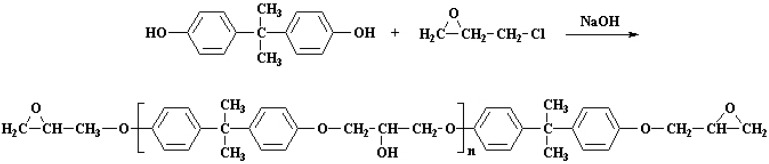
Epoxy prepolymer DGEBA; preparation reaction from bisphenol A (BPA) and epichlorhydrin.

A pre-polymer oligomer usually based on bisphenol A (BPA) is cured with the addition of a curing agent, which can be a variety of compounds such as diamines or polyamines, polyamides and acid anhydrides [5]. Currently, almost 75% of the world production of epoxy prepolymers is produced by the condensation reaction of BPA and epichlorohydrine, yielding the diglycidyl ether of BPA (DGEBA) oligomer/prepolymer, as shown in Figure 1 [6].

The epoxy resins and curing agents commonly used today are derived from petroleum sources. However, pressing environmental concerns, as well as finite petrochemical resources, urge industries towards sustainable products; hence, more research and development methods on bio-based polymers derived from naturally occurring raw materials are needed [7]. These new materials must be tested and developed further to replace conventional ones.

**Figure 2:**
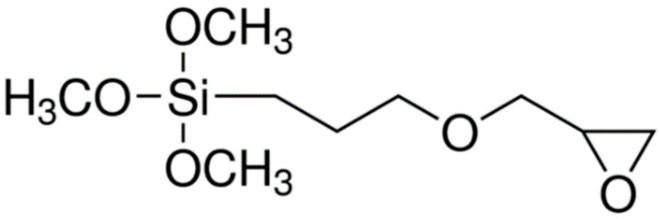
Chemical structure of 3-glycidoxypropyltrimethoxysilane (GLYMO).

The new biobased resins need to be combined with state-of-the art reinforcing agents, such as nanofillers, to meet application requirements. Graphene and graphene related materials (GRMs) have attracted significant attention as advanced carbon nanofillers in polymer matrix nanocomposites [8,9,10]. Graphene flakes can be produced from exfoliation of graphite via solution processing using ultrasonication [11], high shear mixing [12] or microfluidization [13]. Another approach is the production of graphene oxide (GO) from graphite oxide and the subsequent reduction of GO to restore its properties [14,15,16,17,18,19,20,21]. The resultant reduced graphene oxide (rGO) has improved properties compared to the defective GO and contains a small number of oxide groups, which may be beneficial in its interaction with polymer matrices. Improvements in Young’s modulus (*E*) or ultimate tensile strength (*UTS*) of epoxies with the addition of rGO have been reported in [22,23]. To improve further dispersion and interfacial interaction between rGO and resin matrix, which results in better load transfer, certain chemical functionalisations have been reported using organic amines [24], isocyanates [25], azide compounds [26] and organosilane compounds [27,28,29,30,31] such as 3-glycidoxypropyltrimethoxysilane, GLYMO (Figure 2). The epoxy group of GLYMO can react with the epoxy groups of GO to form ether –C–O–C– bonds. Moreover, the hydrolysis of methoxy groups –OCH_3_ results in the formation of silanol groups -Si-OH, which can also react with hydroxyl –OH groups and epoxy groups [29,31]. In addition, GLYMO grafting reduces the hydrophilicity of GO, making it more compatible with the hydrophobic polymer matrix.

An important application area of epoxies is also adhesive bonding offering unique advantages compared to other joining methods, e.g., superior damping, reduced noise, corrosion resistance, reduced stress concentrations and design flexibility. Recently, a few studies reported improvements in adhesion strength of epoxy adhesives with the incorporation of rGO. G. Marami et al. used rGO/Araldite2011 adhesive to bond aluminum alloy 7075-T6 adherents and found that joints with 0.5 wt% rGO exhibited 27% higher lap shear strength (*LSS*) compared to joints connected with neat adhesive [32]. Aradhana et al. used rGO/Araldite GY250 to bond aluminum adherents and showed ~50% increment compared to pristine epoxy [22]. Several studies reported also improvements on epoxies using GO [33] or graphene nanoplatelets [34,35,36,37,38,39].

In the present work, novel nanocomposites with a biobased epoxy/amine system (Epilok 60-600G/Curamine 30-952) reinforced with rGO or GLYMO-rGO were prepared and studied. GRMs contain low oxygen content that aided dispersion into the low viscosity liquid Curamine without the use of a solvent. The cure kinetics of the neat Epilok/Curamine system and the GRM/Curamine/Epilok system were studied by Differential Scanning Calorimetry (DSC) under non-isothermal conditions at different heating rates and under isothermal conditions. The mechanical properties *E* and *UTS* of the neat resin and nanocomposites were determined. CFRP laminates were produced using three layers of carbon fibre sheets (plies) stacked one after another with the same orientation and impregnated with GRM/Curamine/Epilok. These CFRP laminates were used to prepare lap shear joints (CFRP/CFRP and CFRP/Aluminum) and to determine their lap shear strength (*LSS*). It was found that Epilok 60-600G/Curamine 30-952 can be used as a matrix, and the adhesive and addition of only 0.05 wt% GLYMO-rGO in both matrices improved mechanical performance significantly.

## 2. Materials and Methods

### 2.1. Materials

Graphite powder was obtained from NGS-Naturgraphit. Concentrated H_2_SO_4_, KMnO_4_, sodium nitrate, hydrochloric acid and ascorbic acid were purchased from Cofarcas (Burgos, Spain). Epilok 60-600G resin and Curamine 30-952 hardener were provided by Bitrez (Wigan, UK). This is a specially formulated two-part epoxy system designed for the bonding and lamination of composite parts. The system has components derived from biomass, such as plants and/or trees; Epilok 60-600G and Curamine 30-952 have 36% and 66% bio-based renewable content, respectively. Epilok 60-600G has low to moderate viscosity (100–145 Poise evaluated at 25 °C) and mean epoxy equivalent weight (EEW = 185 G/eq). It has CAS number of 25068-38-6, which corresponds to the chemical structure shown in Figure 1. Its average molecular weight is lower than 700.

Curamine 30-952 is a liquid polyaminoamide-based curing agent with a small amount (1–5%) of triethylenetetramine. It has a low-moderate viscosity (100–145 Poise at 25 °C). The Active Hydrogen Equivalent Weight (AHEW) of Curamine 30-952 is 100. It contains 66% bio-based polyaminoamide curing agent. The mix ratio (parts per hundred of epoxy resin) by weight for a stoichiometric ratio is provided by the following relation: mix stoichiometric ratio per 100 parts of epoxy resin = (AHEW of Curamine)/(EEW of epoxy resin) × 100. For the above system, this ratio (Epilok/Curamine) is 100:54. Hοwever, the provider suggests the use of mix ratio 100:85, which is an excess of amine, and this ratio was used in the present work. The amine hardener’s higher level is reported by the manufacturer, which increases the flexibility and toughness of the cured epoxy resin. Carbon Fibre Biaxial Tape 110 mm wide, 410 g per m^2^ with ±45° fibre orientation, was obtained from east coast fiberglass supplies, UK. Aluminium EN AW-7075 T6 was supplied by Alnan Aluminium Co., Ltd., China.

### 2.2. Preparation of GRMs

#### 2.2.1. Preparation of GO

An aqueous suspension of graphite oxide (1.0 wt%) was prepared by using a modified Hummers’ method [16], as described in [40], starting from large flakes of natural graphite and using a proportion of graphite/KMnO_4_/NaNO_3_ 1:3.75:0.25. The reaction temperature inside the reactor was kept between 0 and 4 °C during the oxidant’s addition (reaction time 72 h). The resulting solution was slowly warmed up to 20 °C and maintained for 72 h of reaction. In order to remove excess of MnO_4_-, H_2_O_2_ solution was added to the reaction mixture and stirred overnight. After sedimentation, the solution was washed with HCl 4 wt% solution by 2 h under mechanical stirring. The solid was filtered, obtaining wet graphite oxide. Wet graphite oxide was dispersed in osmotic water (1 wt% based on dry GO) and stirred in a Dispermat LC75 using a cowless helix at 1000 rpm for 10 min and then at 20,000 rpm for 60 s. This dispersion was ultrasonicated with a UP400S HIELCHER ultrasonicator equipped with an H22 sonotrode for 30 min.

#### 2.2.2. Preparation of rGO

rGO was prepared from GO following a thermochemical reduction process [41]. The amount of 4.9 g of ascorbic acid (Vitamin C) used as reducing agent was added into 1 L of the 1 wt% GO water dispersion, and the mixture was refluxed overnight at atmospheric pressure. The solid was filtered off and air-dried. Then, the chemically reduced GO was heated in an oven under Ar atmosphere for 60 min at 200 °C to obtain thermochemically reduced rGO as a black solid with an apparent density of 0.002 g/mL.

#### 2.2.3. Preparation of GLYMO-rGO

The amount of 50 g of rGO was suspended in 1 L of ethanol/water (30/70 *v*/*v*) under stirring to homogenize the suspension. HCl 33% *v*/*v* was added dropwise until the pH was adjusted to 3.5. Then, 50 mL of GLYMO was added and stirred at 60 °C for 24 h. A powder was collected by filtering and washed with water and ethanol 96% *v*/*v* to remove unreacted silane molecules. The obtained powder was placed in an oven and dried at 80 °C for 24 h.

### 2.3. Characterisation of GRM Flakes

Raman spectra were recorded on a confocal Renishaw inVia Raman microscope at room temperature. The system is equipped with a CCD detector and a holographic notch filter using an excitation wavelength of 532 nm. Scans were acquired from 1000 to 3500 cm^−1^, performing maps of 25 spectra on a sample pellet prepared by pressing GRM powder in a 13 mm diameter mould at 5 Tonne/cm^2^. X-ray photoelectron spectroscopy (XPS) analysis was carried out using an ESCAPROBE P (Omnicron, Taunusstein, Germany) with non-monochromatized MgK radiation (1253.6 eV) spectrometer; the X-ray source operated at 300 W. The specific surface area (SSA) of GRMs was determined by Brunauer–Emmett–Teller (BET) using autosorb-6 Quantachrome instruments (Boynton Beach, FL, USA). The samples were degassed in an autosorb degasser (Quantachrome instruments) at 250 °C for 8 h. Transmission electron microscopy (TEM) was performed using a JEOL microscope (JEM-2010) equipped with INCA Energy TEM 100 X-ray detector and a GATAN camera (SC600ORIUS). GRM samples were dispersed in isopropyl alcohol, then sonicated with a Hielscher UP200S sonicator (Teltow, Germany) for 15 min and drop casted onto copper grids. Scanning electron microscopy (SEM) imaging was performed on GRM flakes using a Hitachi S-2400 (18 kV) (Tokyo, Japan)with XFash detector.

### 2.4. Preparation, Characterization and Testing of GRM/Polymer Nanocomposites

GRMs in powder form were first mixed with preheated (50 °C) Curamine using a bath ultrasonicator (Elmasonic P300) for 20 min at 50 °C. Following ultrasonication, Epilok was added via mechanical stirring for 5 min at a ratio Epilok:Curamine of 100:85. The mixture was degassed for 10 min to remove any trapped air. These GRM/Curamine/Epilok nanocomposites were prepared at different GRM content (wt%) and were used for DSC, thermogravimetric analysis (TGA), tensile and lap shear tests, as described in the next section.

#### 2.4.1. DSC Characterisation

The DSC study of the neat epoxy system and GRM/Curamine/Epilok nanocomposites was carried out using a DSC Q10 from ΤA Instruments. The GRM content in these samples was 1.5 wt%. Samples about 10–15 mg were weighted and sealed into aluminium hermetic DSC pans. The sample pan was then placed in the DSC cell previously maintained at room temperature. All DSC runs were carried out under N_2_ atmosphere. Non-isothermal scans were recorded from 20 up to 300 °C with four different heating rates 2, 5, 10 or 20 °C/min. Isothermal scans were recorded at 50, 70 or 90 °C. The DSC cell was quickly heated (50 °C/min) to the desired cure temperature and then isothermally held at that temperature for 3 h. Following this isothermal scan, the DSC cell was immediately cooled down to room temperature and then heated to 300 °C at 10 °C/min to obtain the residual heat of curing. This was determined by integrating over the exothermic peak with respect to time. The total heat of curing recorded isothermally (Δ*H_iso_*) and the residual heat of curing recorded dynamically (Δ*H_dyn_*) were used to determine the degree of curing (*α*) at various isothermal cure temperatures.

#### 2.4.2. Thermogravimetric Analysis

The thermal stability of neat epoxy and prepared nanocomposites was investigated by TGA using a TGA55 thermogravimetric analyser by TA Instruments. Measurements were carried out from 20 °C to 800 °C at a heating rate of 10 °C/min under N_2_ atmosphere.

#### 2.4.3. Tensile Testing

Specimens were prepared for tensile tests (~15 g/per sample) with GRM content 0.05, 0.1, 0.3, 0.5 or 0.7 wt%. Aluminum moulds for tensile specimens were prepared using a CNC milling machine. The GRM/Curamine/Epilok mixture was poured into the moulds, degassed for 10 min in a vacuum chamber and then cured for 10 min at 110 °C in an oven. Dumbbell-shape specimens were obtained at 10 mm wide and 4 mm thick for tensile testing. Tensile tests were performed using a universal testing machine Zwick Roell Z010 with a load cell of 10 kN at room temperature with a crosshead speed of 5 mm/min. The mechanical properties, *E* and *UTS*, were determined according to the ASTM D638 standard.

#### 2.4.4. SEM Characterisation

Specimens were submerged in liquid nitrogen and shattered. Samples were collected and mounted on an aluminium SEM specimen stub by using carbon adhesive discs; stubs and carbon adhesives were purchased from Agar Scientific, UK. A thin metal layer of around 8 nm was sputtered using a Quorum SC7620 Mini Sputter Coater with a gold/palladium target.

#### 2.4.5. Fabrication of CFRP Laminates, Lap Shear Joints and Lap Shear Testing

Laminate specimens (3 plies) were prepared for lap shear testing using ~55 g of neat epoxy or 0.1 wt% GRM/Curamine/Epilok mixture with 55 g carbon fibres/per sheet. The mixture was applied onto each carbon fibre sheet via the wet lay-up process and the 3 plies were cured for 10 min at 110 °C. Rectangular shape specimens were cut 101 mm long and 25.4 mm wide and 1.6 mm thick using water jet cutting to be used as CFRP adherents. These were used to prepare CFRP/CFRP lap joints. CFRP/Aluminium lap joints were also prepared. For the adhesive part of the lap joints, neat epoxy or GRM/Curamine/Epilok was used with GRM content 0.05, 0.1 and 0.5 wt% to bond laminate adherents. CFRP surfaces were rugged with sandpaper P60 and aluminium surfaces with silicon carbide 1200. Then, the surfaces were wiped with a dry cloth to remove particles and cleaned with acetone. The mixture of GRM/Curamine/Epilok was applied on substrate surfaces, bonding them together and cured for 10 min at 110 °C. The joints’ overlapping area was 12.7 mm × 25.4 mm according to the ASTM D5868 standard. The thicknesses of the CFRP laminate and aluminium adherents were 1.6 mm and 2 mm, respectively, and the thickness of the adhesive was 0.2 mm. The joints were tested using a Zwick Roell Z010 at room temperature with a crosshead speed of 5 mm/min.

## 3. Results and Discussion

### 3.1. Characterisation of GRMs

**Figure 3:**
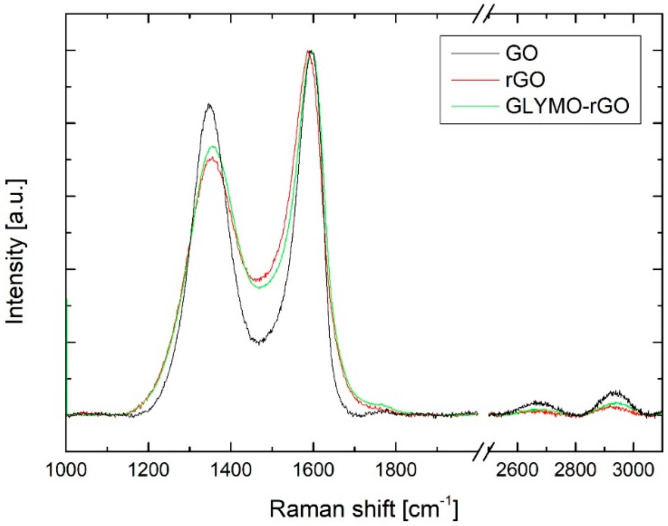
Raman spectra of GO, rGO and GLYMO-rGO.

Figure 3 shows the Raman spectra of the prepared rGO and GLYMO-rGO. It shows an intense D band (~1350 cm^−1^), which confirms lattice distortions, and the G*_app_* band (~1585 cm^−1^), which corresponds to the overlap of G and D’ bands. Two-dimensional band (~2700 cm^−1^) and D+D’ and 2D’ bands, which are different overtone and combination bands of the previous ones, show very small intensity, in alignment with stage 2 defects. GLYMO-rGO shows higher I_D_/I_Gapp_ and lower values of full width at half maximum (FWHM) of D and G peaks when they are compared with the starting rGO (Table 1). This fact is in agreement with a decrease in oxygen content during the functionalisation of rGO [41]. Due to the low intensity in the second order bands in agreement with highly defective rGO materials in Stage 2 defects, any tendency can be observed for I_2D_/I_D_ and I_DD’_/I_2D_.

As mentioned by King et al. [42], the unreliability of the relationship between the I_D_/I_G*app*_ due to the overlap of G and D’ peaks limits the utility of this relationship as a measure of the density of defects in rGO; for that reason, we have combined I_D_/I_G*app*_, FWHM D and FWHM G. A second order transition band 2D’ has been recently used to calculate the inferred energy of D’ (D’inf) and the differences between 2D’ (or D’inf) and Gapp.

Raman spectroscopy findings are also in agreement with those of XPS spectroscopy (Table 2). Higher C/O ratio and lower FWHM of D and G*_app_* were observed for GLYMO-rGO. The relation between D’ − G*_app_* and the C/O ratio obtained by XPS is in good agreement with the data obtained by King et al. and by us for other rGO materials [41] (D’*_inf_* − G*_app_* = 3.89 (rGO) and 7.8 (GLYMO-rGO) C/O 8.1 (rGO) and 12.8 (GLYMO-rGO)). The Si 2p peak originated from GLYMO confirms further the covalent functionalization of rGO with GLYMO. Moreover, the C/O ratio increased in the case of GLYMO because of the reaction of hydroxyl and epoxy groups containing oxygen atoms with groups of rGO.

**Figure 4:**
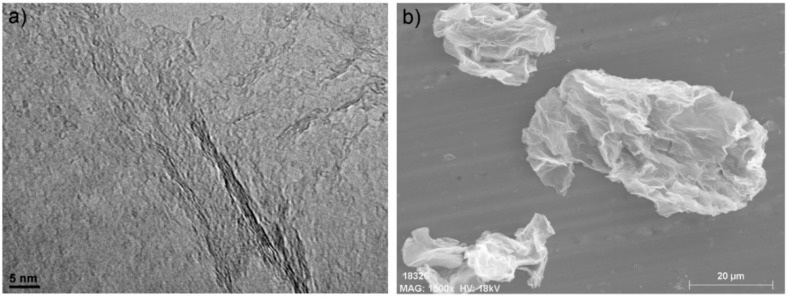
(**a**) TEM image obtained from typical rGO flakes and (**b**) SEM representative image obtained from GLYMO-rGO flakes.

rGO showed high SSA, 778.4 m^2^/g, while GLYMO-rGO showed an important drop in surface area, 146.1 m^2^/g, in agreement with the lower accessible area due to compaction during the filtration process [43], producing non accessible area loss. TEM imaging in Figure 4a obtained from typical rGO flakes revealed low flake thickness in the range of 1 nm, in agreement with the large SSA observed. SEM imaging was used to assess the lateral size of the flakes and was found to be in the 20–50 μm range (Figure 4b). Further characterisation of GRMs, i.e., TEM imaging, X-ray diffraction, XPS spectra and BET isotherms are provided in Supplementary Materials (Figures S1–S5).

### 3.2. Curing Study of GRM/Polymer Nanocomposites by DSC

#### 3.2.1. Curing Mechanism and Kinetics

**Figure 5:**
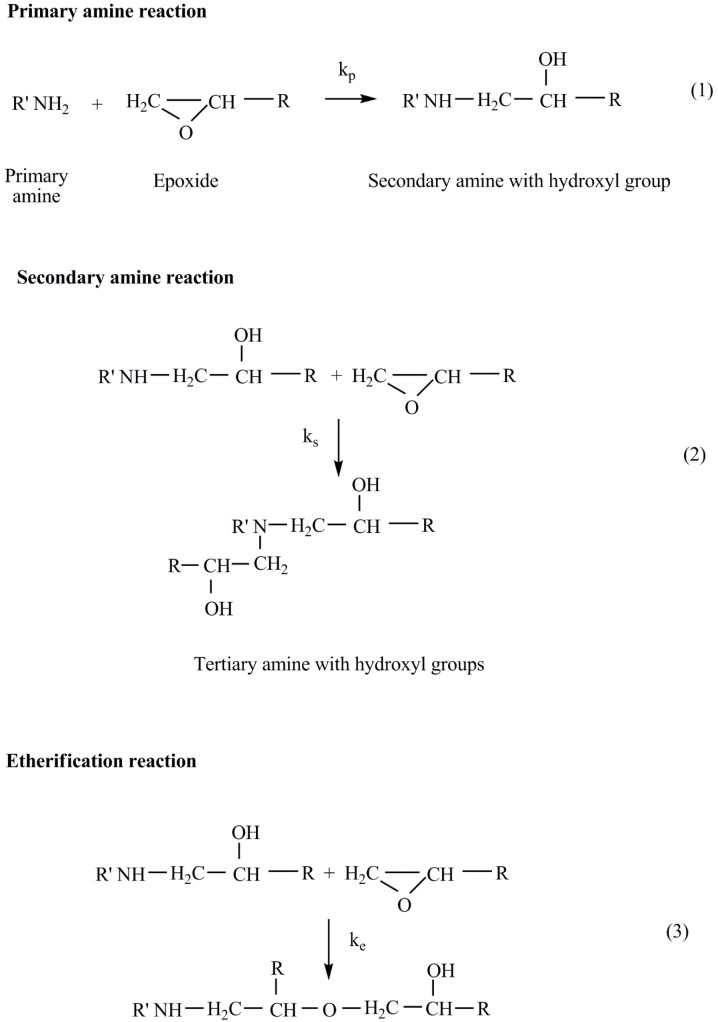
Reactions that may occur during curing an epoxide with a primary amine.

Τhe generally accepted mechanism scheme of the epoxy/primary amine cure involves three main addition reactions of epoxide, as shown in Figure 5 [44]:

For 1:1 epoxy/amine stoichiometry or when amine is present in excess, Reaction (3) is generally insignificant with respect to Reactions (1) and (2). In the majority of systems, the primary and secondary amines have similar reactivities, and one DSC cure exotherm is observed [44], as in the system studied in this work. The kinetic model commonly employed for the epoxy/amine Reactions (1) and (2) was originally derived by Smith [45]. The corresponding reaction scheme (Reactions (4)–(6)) is very simple if the following two assumptions are fulfilled: possible differences in the reactivity of primary and secondary amine can be neglected and intentionally added catalytic species and/or catalytic impurities are missing.

**Figure 6:**
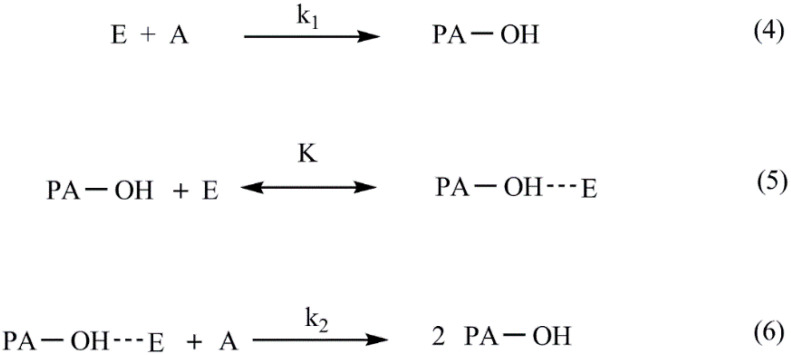
Simplified kinetic model for epoxy/amine reactions (1) and (2).

Where E, A and PA-OH are the epoxide, the amine and the reaction product characterized as polyadduct containing hydroxyl groups, respectively. At the very beginning of the process, the non-catalyzed reaction occurs (Reaction (4)) (primary amine reaction, Figure 6). The formed polyadduct contains hydroxyl groups, which forms hydrogen bonds with the oxygen atom of epoxide (equilibrium Reaction (5)), facilitating the ring opening and reaction with amine [46]. The rate determining step is the autocatalyzed Reaction (6) [47].

Kinetics deal with measurement and parameterization of process rates. DSC is a widely used experimental technique for obtaining a thorough understanding of the cure process. In kinetic analysis by DSC, the rate of reaction is assumed to be proportional to the rate of heat generation and can be expressed as follows:

(1) $$\frac{da}{dt}=\frac{1}{\Delta H_{total}}\left(\frac{dH}{dt}\right)$$

where *α* is the degree of cure, *dα/dt* is the rate of reaction, *dH/dt* is the heat flow and Δ*H_total_* is the exothermic heat expressed as heat per mole of reacting groups (kJ mol^−1^). The degree of curing *α* at any time *t* is given by the following:

(2) $$a\left(t\right)=\frac{\Delta H\left(t\right)}{\Delta H_{total}}$$

where Δ*H(t)* is the heat generated up to time *t.* The ultimate degree of conversion is defined as follows.

(3) $$a_{ult}=\frac{\Delta H_{ult}}{\Delta H_{total}}$$

The majority of kinetic methods used in the area of thermal analysis consider the rate to be a function of only two variables *α* and the absolute temperature *T*.

(4) $$\frac{d\alpha }{dt}=k\left(T\right)f\left(a\right)$$

where *dα/dt* is the rate of reaction, *k(T)* is the temperature dependent rate constant and *f(α)* is the degree of conversion-dependent reaction model.

Equation (4) describes the rate of a single-step process. It is worthy to note that if a process is found to obey a single-step equation, it does not mean that the process mechanism involves one single step. More likely, the mechanism involves several steps, but one of them determines overall kinetics. For instance, this would be the case of two consecutive reactions, when, e.g., the first reaction is significantly slower than the second. Then, the first reaction would determine the overall kinetics that would obey a single-step Equation (4), whereas the mechanism involves two steps [48].

The temperature dependence of the reaction rate is generally defined by using an Arrhenius expression:

(5) $$k\left(T\right)=Ae^{\left(-\frac{E_{a}}{RT}\right)\;}$$

where *A* and *E_α_* are kinetic parameters, the preexponential factor and the activation energy respectively, *R* is the universal gas constant and *T* is the temperature in *K*.

Combining Equations (4) and (5) yields the following.

(6) $$\;\frac{d\alpha }{dt}=A\;e^{\left(-\frac{E_{a}}{RT}\right)}f\left(a\right)$$

This equation is applicable to any temperature program: isothermal or nonisothermal.

#### 3.2.2. Non-Isothermal Curing Scanning Method

The non-isothermal dynamic scanning method involves heating the sample at a constant rate over a desired temperature range. For constant heating rate *φ* in non-isothermal conditions, Equation (6) is frequently rearranged.

(7) $$\varphi \frac{da}{dT}=A\;e^{\left(-\frac{E_{a}}{RT}\right)}\;f\left(a\right)$$

Among various multiple heating rate methods used for determining curing kinetic parameters (*E_α_* and *A*), the most extensively used method is that of Kissinger [49] because of its ease of use in which both *E_α_* and *A* are assumed to be constant [48].

The basic equation of the method has been derived from Equation (6) under the condition of the maximum reaction rate. At this point *d*^2^*α*/*dt*^2^ is zero:

(8) $$\frac{d^{2}a}{dt^{2}}=\left[\frac{E_{a}\varphi }{RT_{p}^{2}}+A\;f{}^{\prime }\left(a_{m}\right)\;e^{\left(-\frac{E_{a}}{RT_{p}}\right)}\right]{\left(\frac{da}{dt}\right)}_{m}=0$$

where *f′(α_m_) = df(α)/dα* and the subscript m denotes the values related to the rate maximum. It follows from Equation (8) that the following is the case.

(9) $$\frac{E_{a}\;\varphi }{R\;T_{p}^{2}}=-A\;f{}^{\prime }\left(a_{m}\right)\;e^{\left(-\frac{E_{a}}{RT_{p}}\right)}$$

After simple rearrangements (Equation (9)) is transformed into the Kissinger equation:

(10) $$ln\left(\frac{\varphi }{{Τ}_{p}^{2}}\right)=ln\left[\left(-\frac{AR}{E_{a}}\right)\;f{}^{\prime }\left(a_{m}\right)\right]-\frac{E_{a}}{RT_{p}}$$

where *T_p_* is the peak temperature. By plotting *ln(φ/Τ_p_^2^)* versus 1*/T_p_*, the values of $E_{a}$ and *A* can be estimated by calculating the slope of the linear fit and the y-intercept.

**Figure 7:**
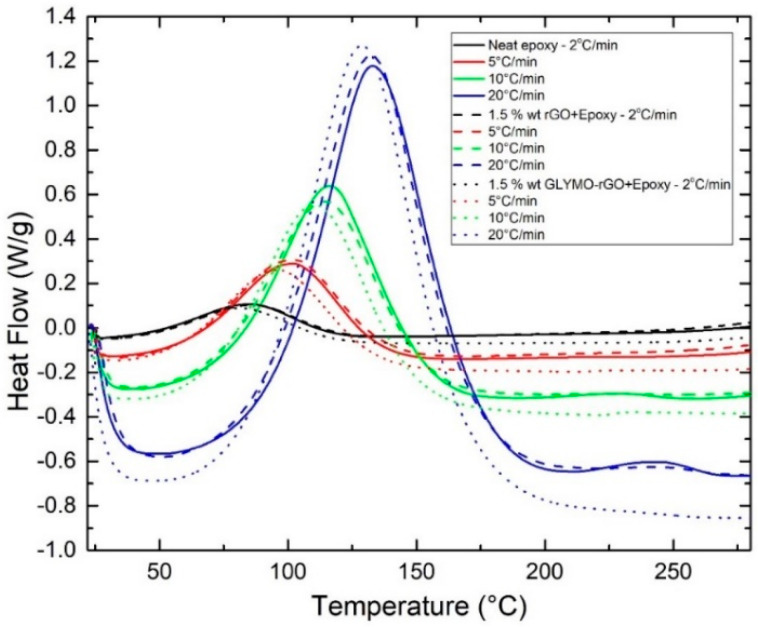
Dynamic scans with different heating rates 2, 5, 10 and 20 °C/min, for neat epoxy and nanocomposites with 1.5 wt% rGO or 1.5 wt% GLYMO-rGO.

It is worth noting that one limitation of the method is associated with the fact that the determination of an accurate $E_{a}$ value requires *f′(α_m_)* to be independent of *φ*. Otherwise, the first term in the right-hand side of equation (10) would not be constant and the plot of *ln(φ/Τ_p_*^2^*)* versus 1*/T_p_* would deviate systematically from a straight line, producing a systematic error in $E_{a}$ [48]. In Figure 7, the dynamic scans with different *φ*, 2, 5, 10 and 20 °C/min, for neat epoxy with 1.5 wt% rGO or 1.5 wt% GLYMO-rGO are shown.

The results obtained for the initial cure (or onset) temperature (*T_init_*), peak cure temperature (*T_p_*), final cure temperature (*T_final_*), ultimate heat of curing (Δ*H_ult_*) and degree of curing (*α*) for the studied samples under different heating rates are presented in Table 3. It is observed that, for all systems studied, *T_init_*, *T_p_* and *T_final_* increased with increasing φ, because lower *φ* offered longer time periods for chemical groups to react, while at faster *φ* less time is needed for the reaction of the groups.

The DSC curves shift to higher temperatures to compensate for the reduced time [50]. *Τ_p_* of 1.5 wt% rGO or GLYMO-rGO is slightly lower than *T_p_* of neat epoxy. The ultimate heat of curing Δ*H* (J/g) was determined by integration of the exothermic peak. Taking into account that we have used a ratio of 100 g epoxy resin to 85 g of amine, 1 g of the mixture contains 0.540 g of epoxy resin, and the value of Δ*H_ult_* (J/g)_epoxy_ was calculated from the experimental value (J/g)_mixture_ divided by 0.540. Since the EEW of epoxy resin used is 185 g/eq (e.g., 185 g of Epilok 60–600G is 1 gmol of epoxy groups), the value of Δ*H_ult_* (kJ/mol)_epoxy_ was calculated by Δ*H_ult_* (kJ/g)_epoxy_ × 185. Δ*H_total_* measured for several amine-epoxy systems and model reactions was found to be reasonably constant and equal to 107 ± 4 kJ mol of epoxide; this value may be used as a standard value for analyzing amine-epoxide systems [44,51]. The values of *E_a_* were determined graphically by the Kissinger method, as described in our previous communication [52]. *E_a_* is the minimum energy requirement that must be met for a chemical reaction to occur. *E_a_* was found for the neat epoxy to be 49.68 ± 1.65 kJ/mol. The addition of 1.5 wt% of rGO and rGO-GLYMO did not have any significant effect on *E_a_* with values 53.88 ± 3.34 and 49.48 ± 2.89 kJ/mol, respectively.

#### 3.2.3. Isothermal Scanning Method

**Figure 8:**
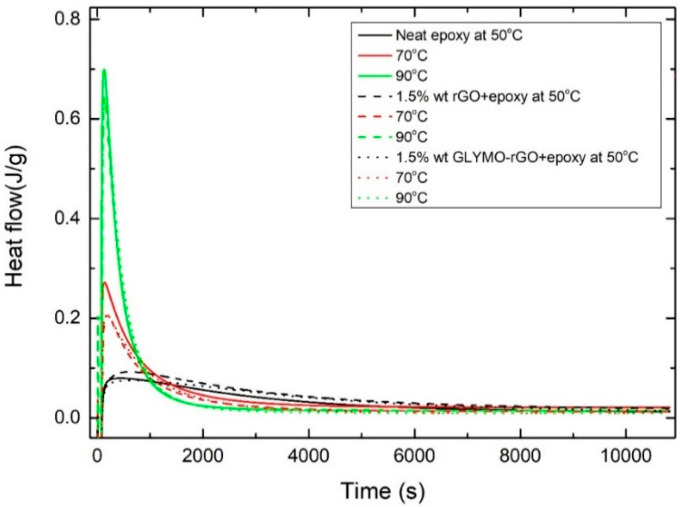
Isothermal DSC thermograms for neat epoxy and nanocomposites containing 1.5 wt% rGO at different temperatures.

**Figure 9:**
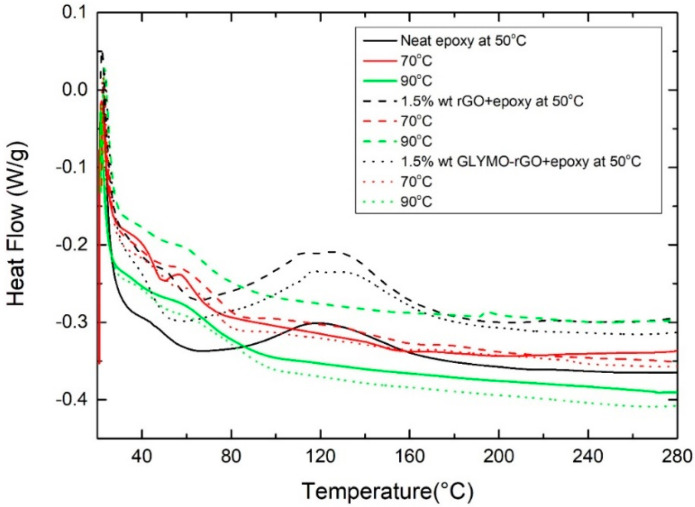
Subsequent non-isothermal DSC scans at a constant heating rate of the partially cured samples derived from the isothermal scans.

The isothermal DSC curves obtained at 50, 70 and 90 °C and the subsequent dynamic scans are shown in Figure 8 and Figure 9, respectively. From these curves, the total curing enthalpy Δ*H_iso_* at a certain temperature and time was determined (Table 4). Following isothermal scans, the samples immediately cooled at room temperature and then heated to 300 °C at 10 °C/min, and the dynamic Δ*H_dyn_* were determined. From the data shown in Table 4, a relative degree of conversion *α_1_* can be calculated as follows.

(11) $$\alpha_{1}=\frac{\Delta H_{iso}}{\Delta H_{iso}+\Delta H_{dyn}}$$

The degree of conversion *α_2_* (Table 4) was calculated from Equation (3) using Δ*H_tot_* of 107 kJ/mol being the average enthalpy for primary and secondary amine reactions [53].

All samples isothermally cured at 90 °C did not show any residual enthalpy in the subsequent dynamic scan. As expected, *α*_1_, *α*_2_ and *T_g_* increased with the increase in curing temperature. It can be observed that both *α* and *T_g_* increased with the addition of GRM. For example, at 90 °C isothermal curing, the degree of conversion *α*_2_ increases from 83.6% for the neat resin to 86.1% and 93.4% with the addition of 1.5 wt% rGO and GLYMO-rGO correspondingly due to the higher crosslinking density. Moreover, at 90 °C isothermal curing, *T_g_* increases from 83.6 °C for the neat resin to 94.2 °C and 97.5 °C with the addition of 1.5 wt% rGO and GLYMO-rGO, correspondingly improving thermal stability.

### 3.3. Thermogravimetric Analysis

**Figure 10:**
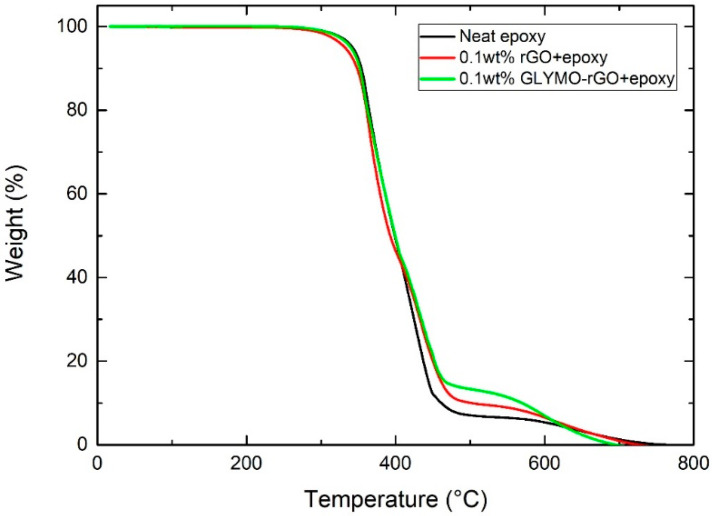
TGA spectra obtained from neat epoxy and nanocomposites containing 0.1 wt% rGO and 0.1 wt% GLYMO-rGO.

Figure 10 presents TGA curves of the epoxy and the nanocomposites containing 0.1 wt% rGO and 0.1 wt% GLYMO-rGO. The initial decomposition temperature, which corresponds to 5% weight loss [33], was found to decrease from 342.2 °C for neat epoxy, 331.91 °C for 0.1 wt% rGO and 339.7 °C for 0.1 wt% GLYMO-rGO. This could result from the early decomposition of interfacial epoxy chains, the cure of which was partially inhibited by the inclusion of the nanofiller [33]. Dominant weight loss occurs above 350 °C due to the thermal decomposition of epoxy resin. The temperature of the maximum rate of degradation decreases from 394.82 °C of the neat epoxy resin to 386.04 °C for 0.1 wt% rGO and 381.78 for GLYMO-rGO. The TGA curve of the nanocomposites was shifted in the range of 408–615 °C towards higher temperature compared to that of pure epoxy increasing the thermal stability of cured epoxy nanocomposites. The percentage weight residue at 500 °C is 7.1% for the neat resin, 10% for rGO and 13.3% for GLYMO-rGO.

### 3.4. Mechanical Properties

#### 3.4.1. Tensile Testing GRM/Polymer Nanocomposites

**Figure 11:**
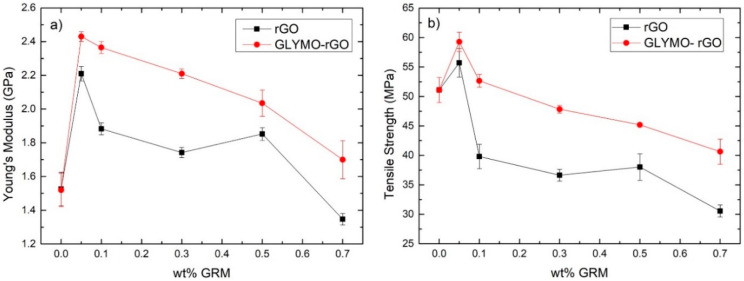
Mechanical properties of cured nanocomposites reinforced with different amounts of GRMs; (**a**) Young’s modulus; (**b**) ultimate tensile strength.

The results obtained from tensile tests are shown in Figure 11. The addition of only 0.05 wt% of rGO increased *E* from 1.52 GPa to 2.21 GPa and *UTS* from 51.1 MPa to 55.71 MPa, an increase by 45.39% and 9.02%, respectively. The addition of 0.05 wt% of GLYMO-rGO increased *E* to 2.43 GPa and *UTS* to 59.27 Mpa, an increase by 59.86% and 15.98%, respectively. This increase is due to chemical reactions between the functional groups of GRMs with the epoxy matrix resulting in covalent bonding of rGO and GLYMO-rGO groups with the epoxy matrix, causing effective load transfer and enhanced mechanical properties at such low GRM content [29]. In both GRMs, *E* and *UTS* decreased at higher filler content probably due to poor dispersion of GRMs in the epoxy matrix [29]. A comparison of the results of this work with other literature reporting on rGO/epoxy nanocomposites is provided in Table S1.

#### 3.4.2. Microscopical Investigation

**Figure 12:**
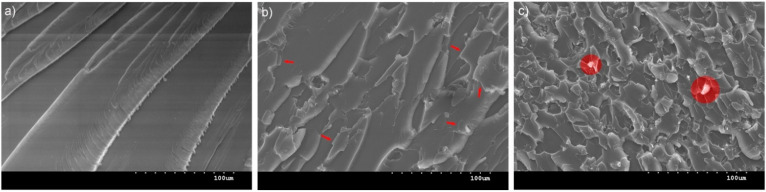
SEM images of fractured surfaces of (**a**) neat epoxy, (**b**) nanocomposite with 0.05 wt% rGO and (**c**) nanocomposite with 0.1 wt% rGO.

In order to understand the mechanisms of failure, the fracture surfaces were investigated using SEM. The neat epoxy resin exhibited a featureless image typical of brittle fracture surface (Figure 12a). The addition of 0.05 wt% rGO (Figure 12b) shows a different morphology with a rougher surface, which indicates that higher energy was consumed to create a larger fractured surface [32]. This morphology is in agreement with other rGO/epoxy reports [22], which suggest that when a crack front encounters any rigid rGO inclusion indicated with arrows, it deviates from its original path generating more fracture surface area. Nanocomposites with 0.1 wt% rGO shown in Figure 12c present an even rougher surface. However, agglomerated clusters indicated with circles are present, which should be responsible for the decrease in mechanical properties as these can act as stress concentration points for initiating rupture [22]. Olowojoba et al. [23] found agglomeration to be present above 0.06 wt%, which is in agreement with our results.

#### 3.4.3. Lap Shear Joints with CFRP/CFRP or CFRP/Aluminium Laminate Adherents

**Figure 13:**
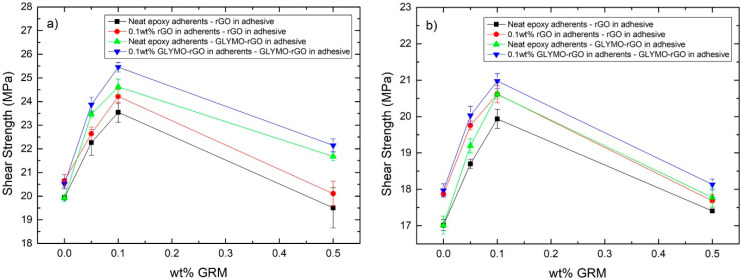
Lap shear strength versus wt% of GRM in adhesive for (**a**) CFRP/CFRP adherents and (**b**) CFRP/Aluminum adherents.

Lap shear joints were prepared and tested according to the ASTM D5868 standard test method for lap shear adhesion of fibre-reinforced plastic bonding. *LSS* was calculated from the peak load divided by the shear area of the joint. Figure 13a shows *LSS* versus wt% of the GRM added in the adhesive or adhesive and CFRP adherents. It is shown that the addition of 0.1 wt% rGO in the adhesive in joints with adherents made of neat epoxy increased *LSS* from 19.92 to 23.54 MPa (18.17% increase). Interestingly, the addition of 0.1 wt% rGO in the adhesive and CFRP adherents improved *LSS* further to 24.24 MPa (21.53% increase compared to neat). When GLYMO-rGO (0.1 wt%) was used in the adhesive only, *LSS* increased from 19.92 to 24.62 MPa (23.59% increase). The highest *LSS* of 25.45 MPa was observed when 0.1 wt% GLYMO-rGO was added into both the adhesive and adherents, which corresponds to a 24.08% increase compared to neat reference samples. Improvements in *LSS* can be attributed to better interaction between the adhesive and adherent, which increased mechanical interlocking and chemical bonding between the GRM and epoxy matrix. Consistent with tensile results, the higher concentration of GRM produced lower *LSS* possibly due to the agglomeration of GRM.

In the case of joints made with CFRP/Aluminium adherents a similar trend is observed with samples showing the highest *LSS* at 0.1 wt% of GRM in the adhesive and CFRP adherent (Figure 13b). The highest *LSS* obtained was 20.97 MPa with 0.1% of GLYMO-rGO, an improvement by 23.28% compared to the reference CFRP/Aluminium sample without GRM. *LSS* is lower in CFRP/Aluminium joints compared to CFRP/CFRP joints due to the weaker adhesive/Aluminium interface.

**Figure 14:**
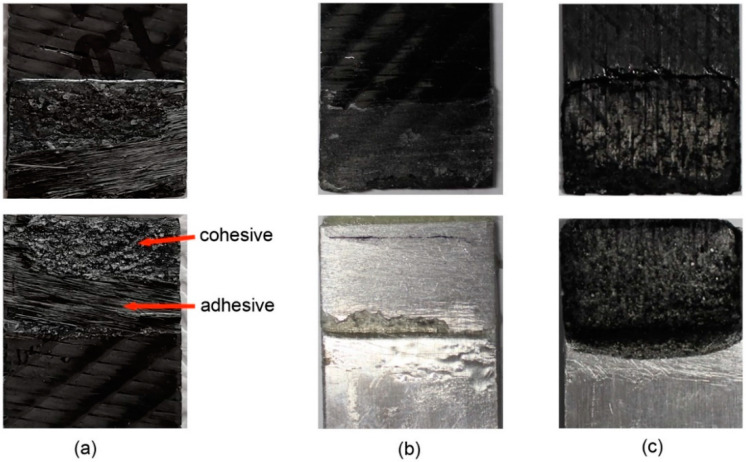
Representative fracture surfaces of (**a**) CFRP/CFRP joints, (**b**) CFRP/Aluminium with neat adhesive and (**c**) CFRP/Aluminium with 0.1 wt% GLYMO-rGO in adhesive.

After shear lap testing, the fractured surfaces were investigated to elucidate failure modes. In CFRP/CFRP samples, the typical failure was a combination of adhesive and cohesive modes, as shown in Figure 14a. In CFRP/Al samples, when a neat adhesive was used, adhesive failure was observed at the Aluminium/adhesive interface (Figure 14b). The addition of GRM improved the adhesive/Al interface and *LSS*. Figure 14c shows cohesive failure with both adherents retaining the adhesive, which was reinforced with 0.1 wt% GLYMO-rGO.

## 4. Conclusions

In the present work, we successfully synthesised GRMs (rGO and GLYMO-rGO). Subsequently, we prepared new GRM/biobased epoxy nanocomposites and studied their cure kinetics, mechanical properties and bonding performance. It was found that the addition of 1.5 wt% of GLYMO-rGO into the epoxy increases the degree of cure by up to 12% and the glass transition temperature by 14 °C. The GRMs also improved the thermal stability of the epoxy system in the range of 408–615 °C. The addition of only 0.05 wt% GLYMO-rGO improves Young’s modulus and tensile strength by 60% and 16%, respectively, compared to neat epoxy. The GRM-enhanced resin was used as a matrix system to prepare CFRP laminates, as well as adhesive to prepare lap shear joints, CFRP (similar adherents) and CFRP/Aluminum (dissimilar adherents). The addition of 0.1 wt% GLYMO-rGO into the adhesive and CRFP adherents showed improved lap shear strength by 23.6% compared to the neat resin, while in the case of CFRP/Aluminium joints the increase was 21.2%.

## Figures and Tables

**Table 1:** Characteristic Raman peaks and intensity ratios for the GRMs used.

GRM	D (cm^−1^)	G_app_ (cm^−1^)	I_D_/I_Gapp_	FWHM_D_	FWHM_G_	I_2D_/I_D_	I_DD’_/I_2D_	D’*_inf_*	D’*_inf_* − G*_app_*
rGO	1363.9	1581.5	0.68	180.3	101.8	0.015	2.29	1588.7	3.89
GLYMO-rGO	1359.6	1588.0	0.74	146.6	92.8	0.010	2.44	1595.8	7.80

**Table 2:** Results obtained by XPS spectroscopy for GRMs.

GRM	C 1s (%)	O 1s (%)	Si 2p (%)	C/O
rGO	88.7	11.0	-	8.1
GLYMO-rGO	92.5	7.2	0.45	12.8

**Table 3:** Characteristic kinetic parameters of curing process: characteristic temperatures, exothermic heat of curing (Δ*H*) and degree of curing (*α*) obtained under non-isothermal conditions with different heating rates *φ*.

GRM (wt%)	*φ* (°C/min)	*T_init_* (°C)	*T_final_* (°C)	*T_p_* (°C)	Δ*H_ult_* ^a^ (J/g)	Δ*H_ult_* ^b^ (kJ/mol)	*α* (%)
0 (neat epoxy)	2	28.72	276.52	83.10	173.35	59.38	55.5
	5	30.12	283.06	100.56	243.62	83.46	78.0
	10	33.98	281.96	114.53	275.57	94.40	88.2
	20	42.025	258.16	132.83	275.80	94.48	88.3
1.5 rGO	2	27.77	278.22	84.71	193.98	66.45	62.1
	5	32.46	277.66	99.49	243.72	83.49	78.0
	10	36.74	284.59	112.78	268.47	91.97	86.0
	20	44.75	283.19	130.84	287.36	98.44	92.0
1.5 GLYMO-rGO	2	28.29	276.02	79.07	212.13	72.67	68.0
	5	30.15	284.91	93.99	276.61	94.76	88.6
	10	33.98	283.92	109.12	290.26	99.44	92.9
	20	37.74	287.78	127.35	312.32	106.99	100

^a^. Δ*H_ult_* (J/g) refers to 1 gr mixture of epoxy determined from the experimental value divided by 0.540. ^b^. Δ*H_ult_* (kJ/mol) calculated from Δ*H_ult_* (kJ/g) × 185 (=EEW of Epilok 60-600G).

**Table 4:** Exothermic heat of curing during the isothermal scans (Δ*H_iso_*), during subsequent dynamic scans (Δ*H_dyn_*), degree of curing (*α*) and glass transition temperature (*T_g_*).

GRM (wt%)	*T* (°C)	Δ*H_so_* (J/g)	Δ*H_iso_* (kJ/mol)	Δ*H_yn_* (J/g)	Δ*H_dyn_* (kJ/mol)	*α_1_* (%)	*α_2_* (%)	*T_g_* (°C)
0 (neat epoxy)	50	209.16	71.65	35.22	12.06	85.6	66.7	53.1
	70	214.37	73.44	0.120	0.041	99.8	68.6	73.3
	90	261.21	89.48	0	0	100	83.6	83.6
1.5 rGO	50	242.63	83.12	26.09	8.92	96.5	77.7	64.5
	70	255.86	87.65	0.24	0.082	99.9	81.9	84.2
	90	268.94	92.13	0	0	100	86.1	94.2
1.5 GLYMO-rGO	50	243.30	83.35	24.35	8.33	96.7	77.9	55.9
	70	260.96	89.40	0.318	0.108	99.9	83.6	83.4
	90	291.66	99.92	0	0	100	93.4	97.5

## Data Availability

The raw/processed data required to reproduce these findings cannot be shared at this time as the data are a part of an ongoing study.

## Supplementary Materials

The following are available online at https://www.mdpi.com/article/10.3390/nano12020222/s1, Figure S1: TEM micrographs obtained from rGO flakes, Figure S2: TEM micrographs of GLYMO-rGO, Figure S3: X-ray diffraction patterns obtained from GO, rGO and GLYMO-rGO flakes, Figure S4: (a) XPS spectrum of GLYMO-rGO, (b) high resolution deconvoluted O1s and (c) deconvoluted C1s spectra of GLYMO-rGO, Figure S5: BET isotherms for rGO and GLYMO-rGO, Figure S6: Representative stress-strain curves obtained from neat epoxy and nanocomposites. Table S1: Literature data of studies on rGO/epoxy nanocomposites and their mechanical properties. References [54,55,56] were cited in Supplementary Materials.
