# Supplementary material for: Βio-Based Epoxy/Amine Reinforced with Reduced Graphene Oxide (rGO) or GLYMO-rGO: Study of Curing Kinetics, Mechanical Properties, Lamination and Bonding Performance
Source: Nanomaterials (Basel). 2022 Jan 10;12(2):222. doi: 10.3390/nano12020222 (PMC8778273; doi:10.3390/nano12020222)
Supplement: Supplementary file 1 [file nanomaterials-12-00222-s001.zip › nanomaterials-1500171-supplementary.pdf]

nanomaterials

Supplementary Materials

Bio-based epoxy/amine reinforced with reduced graphene oxide (rGO) or GLYMO-rGO: study of curing kinetics, mechanical properties, lamination and bonding performance

Sheikh Rehman [1], Julio Gomez [2], Elvira Villaro [3], Dwane Cossey [1] and Panagiotis G. Karagiannidis [1,*]

[1] School of Engineering, Faculty of Technology, University of Sunderland, Sunderland SR6 0DD, UK; bh31lt@research.sunderland.ac.uk (S.R.); bg72yy@student.sunderland.ac.uk (D.C.)
[2] Avanzare Innovacion Tecnologica S.L. Av. Lentiscares 4-6, 26370 Navarrete, Spain; julio@avanzare.es
[3] Instituto de Tecnologías Químicas de La Rioja (Inter-Química), San Francisco 11, 26370 Navarrete, Spain; evillaro@interquimica.org
* Correspondence: Panagiotis.Karagiannidis@sunderland.ac.uk

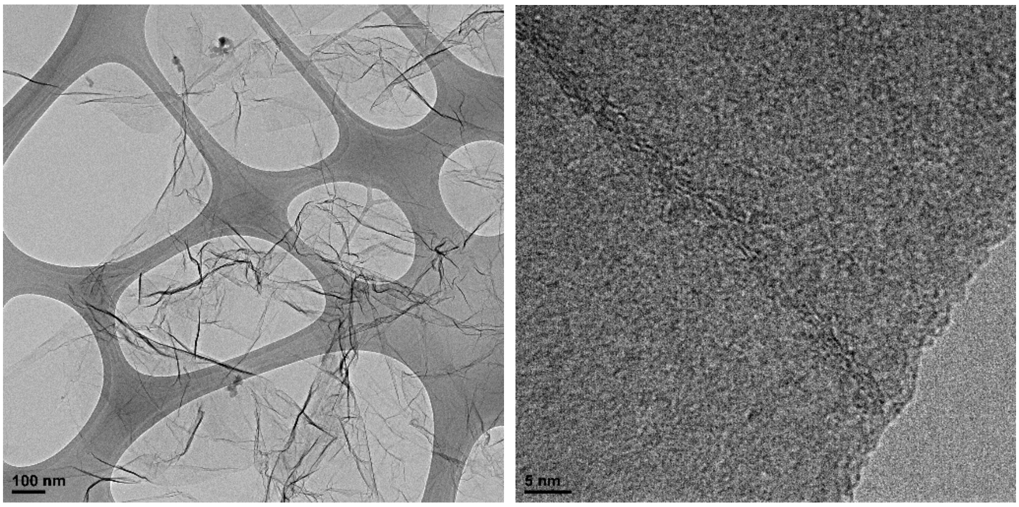

Figure S1. TEM micrographs obtained from rGO flakes.

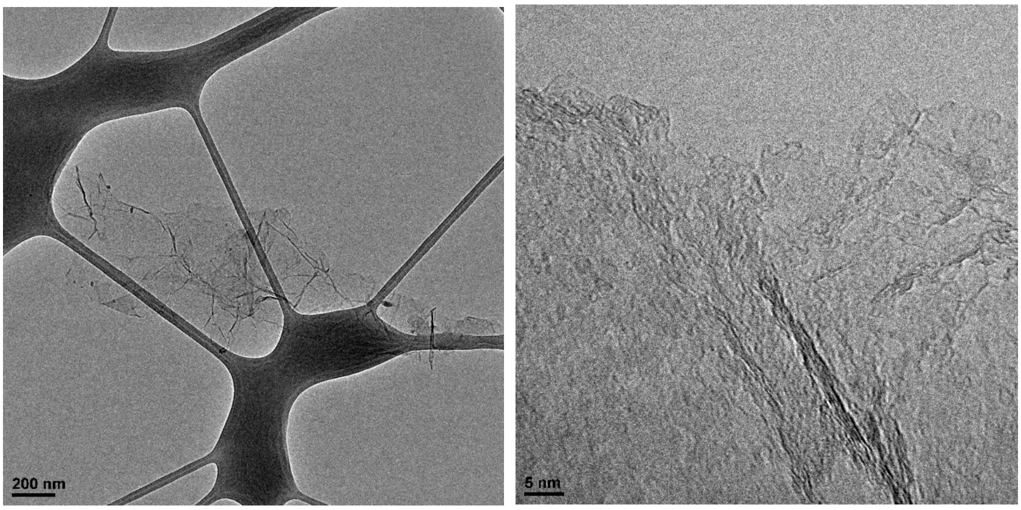

Figure S2. TEM micrographs of GLYMO-rGO.

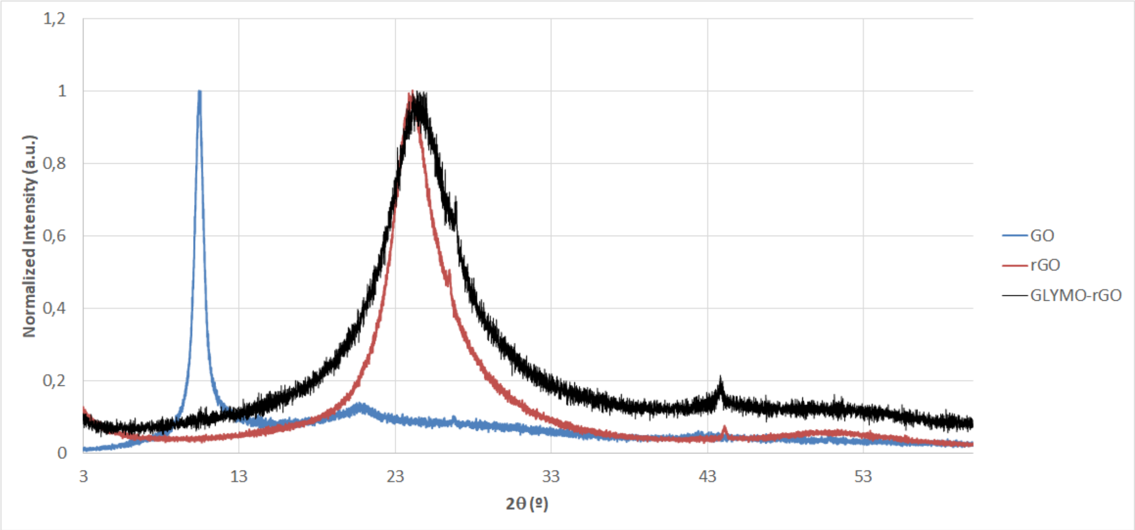

Figure S3. X-Ray diffraction patterns obtained from GO, rGO and GLYMO-rGO flakes.

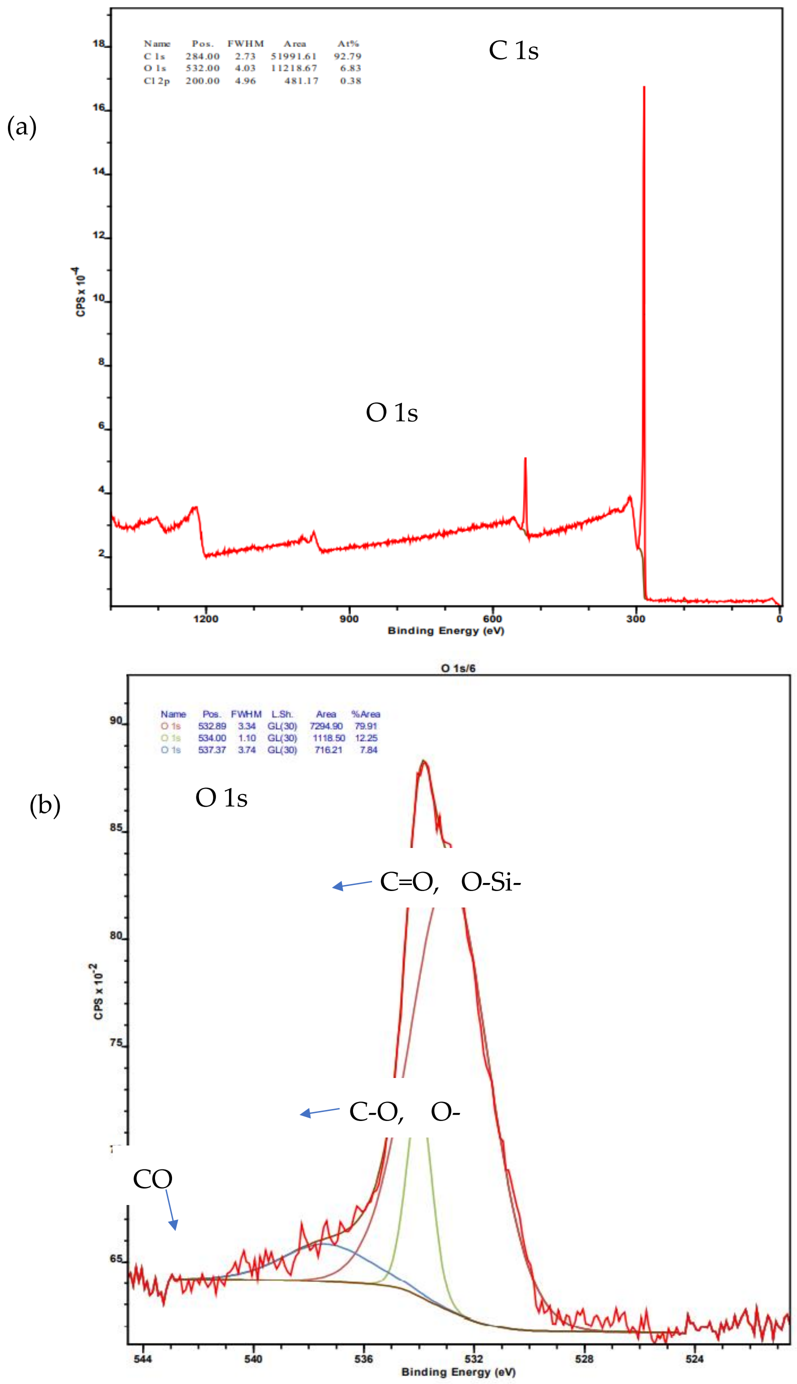
(a)
Name Pos. FWHM Area At%
C 1s 284.00 2.73 51991.61 92.79
O 1s 532.00 4.03 11218.67 6.83
Cl 2p 200.00 4.96 481.17 0.38
C 1s
O 1s
CPS x 10-4
Binding Energy (eV)
(b)
O 1s/6
Name Pos. FWHM L.Sh. Area %Area
O 1s 532.89 3.34 GL(30) 7294.90 79.91
O 1s 534.00 1.10 GL(30) 1118.50 12.25
O 1s 537.37 3.74 GL(30) 716.21 7.84
O 1s
C=O, O-Si-
C-O, O-
CO
CPS x 10-2
Binding Energy (eV)

(c)

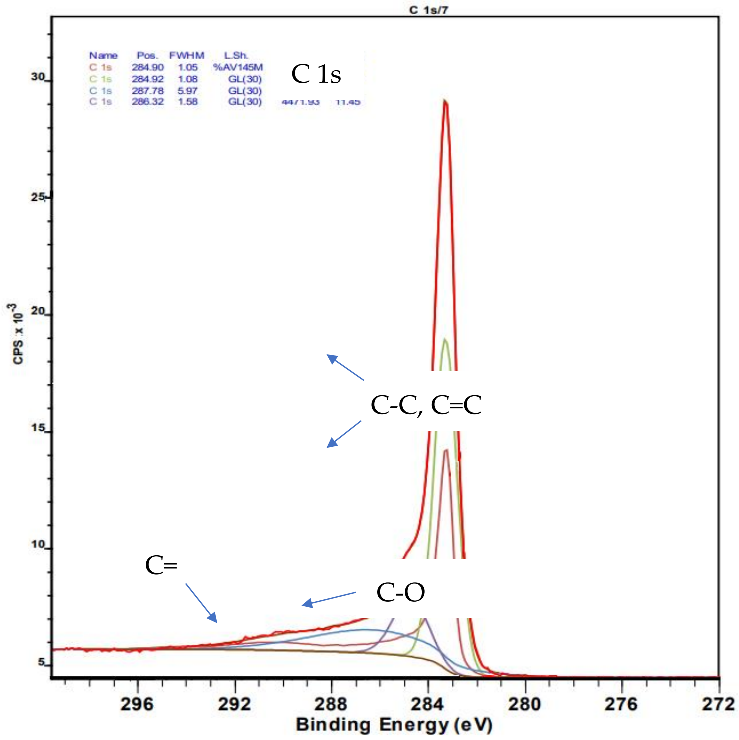

Figure S4. (**a**) XPS spectrum of GLYMO-rGO, (**b**) high resolution deconvoluted O_{1s} and (**c**) deconvoluted C_{1s} spectra of GLYMO-rGO.

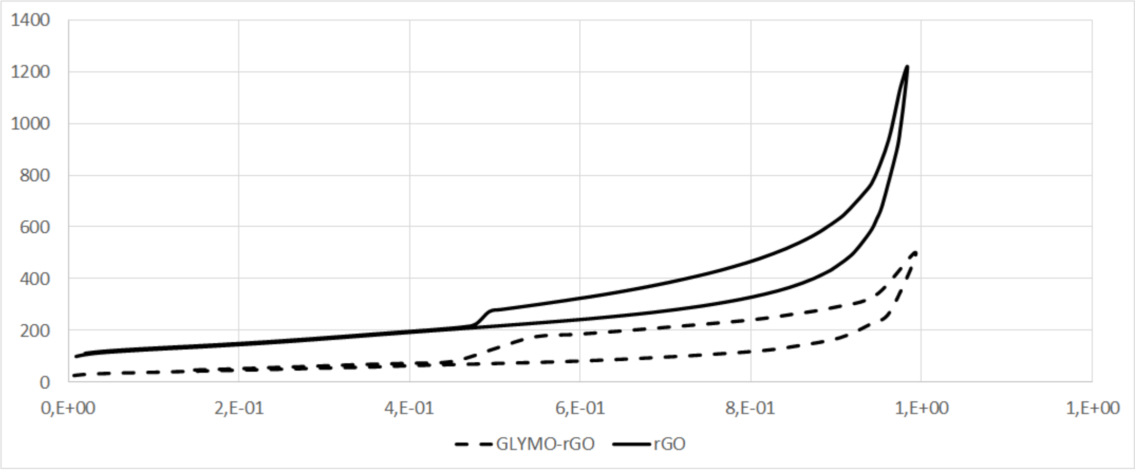

Figure S5. BET isotherms for rGO and GLYMO-rGO.

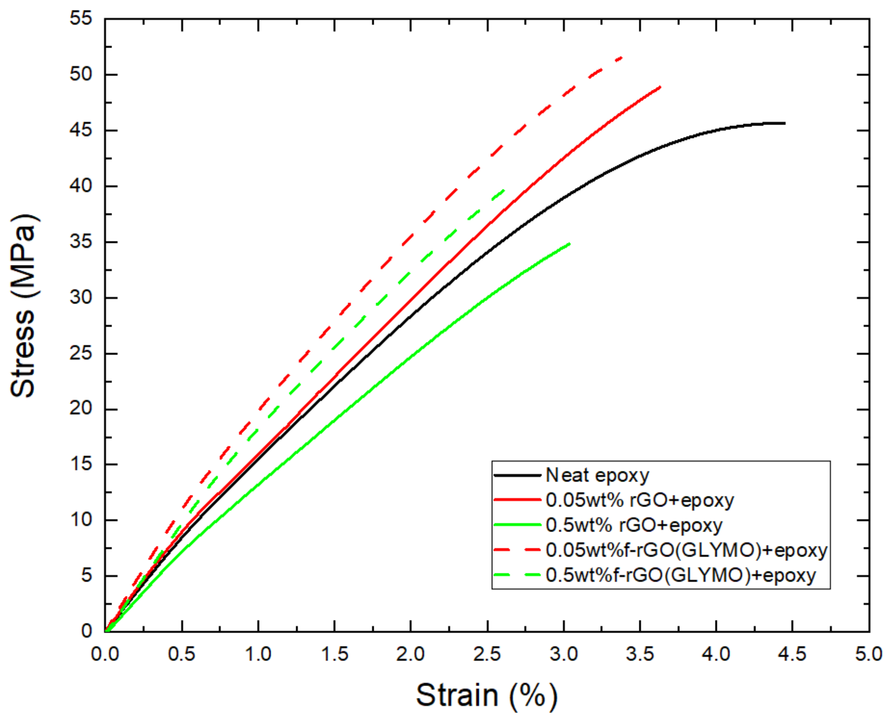

Figure S6. Representative stress-strain curves obtained from neat epoxy and nanocomposites.

Table S1. Literature data of studies on rGO/epoxy nanocomposites and their mechanical properties.

Reference	Nanocomposite Preparation Method	Solvent	Optimum Filler Content (wt%)	Elastic Modulus [GPa] (% Change)	Tensile Strength [MPa] (% Change)
Ramos-Galicia et al. [1]	ultrasonication	-	0.4	1.2 (20% increase)	31 (0% change)
Peng et al. [2]	mechanical stirring in-situ thermal reduction	water	0.43	12.1 ± 0.6 (86% increase)	83.7 ± 8 (51% increase)
Olowojoba et al. [3]	three-roll mill, in-situ thermal reduction	water	2	3.29 (13.44% increase)	44.1 (35.8% decrease)
Olowojoba et al. [4]	mechanical stirring, in-situ thermal reduction	water	0.06	3.11 (7.24% increase)	10 (85.4% decrease)
Aradhana et al. [5]	mechanical stirring, ultrasonication	acetone	0.5	3.3 ± .076 (27.8% increase)	62.02 ± 7.6 (30.70% increase)
This work					
rGO/epoxy	ultrasonication, mechanical stirring	-	0.05	2.21 (45.39% increase)	55.71 (9.02% increase)
GLYMO-rGO/epoxy		-	0.05	2.43 (60% increase)	59.27 (16% increase)

References

[1] Ramos-Galicia L., Mendez L.N., Martínez-Hernández A.L., Espindola-Gonzalez A., Galindo-Esquivel I.R., Fuentes-

Ramirez R, et al. Improved Performance of an Epoxy Matrix as a Result of Combining Graphene Oxide and Reduced Graphene. *Int. J. Polym. Sci.* **2013,** 493147, https://doi.org/10.1155/2013/493147.

[2] Peng M., Tang X., Zhou Y. Fast phase transfer of graphene oxide from water to triglycidyl para-aminophenol for epoxy composites with superior nanosheet dispersion. *Polymer,* **2016**, *93,* 1–8. https://doi.org/https://doi.org/10.1016/j.polymer.2016.03.016.

[3] Olowojoba GB, Eslava S, Gutierrez ES, Kinloch AJ, Mattevi C, Rocha VG, et al. In situ thermally reduced graphene oxide/epoxy composites: thermal and mechanical properties. *Appl. Nanosci.* **2016**, *6,* 1015–22. https://doi.org/10.1007/s13204-016-0518-y.

[4] Olowojoba GB, Kopsidas S, Eslava S, Gutierrez ES, Kinloch AJ, Mattevi C, et al. A facile way to produce epoxy nanocomposites having excellent thermal conductivity with low contents of reduced graphene oxide. *J. Mater. Sci.* **2017**, *52*, 7323–44, https://doi.org/10.1007/s10853-017-0969-x.

[5] Aradhana R, Mohanty S, Nayak SK. Comparison of mechanical, electrical and thermal properties in graphene oxide and reduced graphene oxide filled epoxy nanocomposite adhesives. *Polymer,* 2018, *141,* 109–23, https://doi.org/https://doi.org/10.1016/j.polymer.2018.03.005.
